# Smartphone Use and Physical Activity among College Students in Health Science-Related Majors in the United States and Thailand

**DOI:** 10.3390/ijerph16081315

**Published:** 2019-04-12

**Authors:** Nattika Penglee, Richard W. Christiana, Rebecca A. Battista, Ed Rosenberg

**Affiliations:** 1 Department of Physical Education, Kasetsart University, Bangkok 10900, Thailand; fedunkp@ku.ac.th; 2 Department of Health and Exercise Science, Appalachian State University, Boone, NC 28608, USA; battistara@appstate.edu; 3 Department of Sociology, Appalachian State University, Boone, NC 28608, USA; rosenberge@appstate.edu

**Keywords:** exercise, technology, young adults

## Abstract

Smartphone use among college students is prevalent across the world. Recently, research has begun to investigate the relationship between smartphone use and physical activity. This study examined the amount of time spent using a smartphone and the physical activity (PA) levels among college students majoring in health science-related disciplines in the United States (US) and Thailand. Using convenience sampling, college students in the US (*n* = 242) and Thailand (*n* = 194) completed an online survey, in Fall 2016, assessing smartphone usage and PA. Data were analyzed using chi-square tests and two-way ANOVA (*p* < 0.05). US students reported more days per week (U=15,150.0,p=0.00,r=0.33) and greater duration of PA (U=11,234.0,p=0.00,r=0.33) than Thai students while Thai students used smartphones more per day than US students (U=13,137.5,p=0.00,r=0.40). No difference existed for years of smartphone use (U=22,207.0,p=0.27). Greater smartphone use per day inversely related to days per week of engaging in PA among Thai students ($X^{2}\left(3\right)=10.55,p=0.01,\varepsilon^{2}=0.06$), but not among US students ($X^{2}\left(3\right)=2.39,p=0.50$). The high smartphone use among college students, especially in Thailand, may be a barrier to PA as well as a strategy for PA promotion in higher education settings. Research should examine the best techniques for smartphone application development to promote PA in college settings.

## 1. Introduction

Physical activity has long been recognized as a key dimension of a healthy lifestyle [1]. Research has provided strong evidence that continually engaging in recommended levels of physical activity is associated with numerous health benefits [2,3]. These include reducing the risk of cardiovascular disease, stroke, type II diabetes, certain types of cancer, and reduced symptoms of depression. The World Health Organization has recommended that adults age 18–64 years engage in at least 150 min of moderate-intensity physical activity or at least 75 min of vigorous-intensity physical activity or an equivalent combination of moderate- and vigorous-intensity physical activity per week for reduced risk of various chronic diseases [3]. As such, many countries realize the benefits of physical activity and therefore encourage their citizens to incorporate more physical activity into their daily lives [3].

Despite the increasing efforts of public health professionals, the levels of physical activity among children and adults in the United States (US) and Thailand remain low [4,5,6,7]. In the United States, only 48.7% of college students meet the World Health Organization’s recommended guidelines for physical activity (at least 150 min of moderate-intensity physical activity throughout the week, or at least 75 min of vigorous-intensity physical activity throughout the week, or an equivalent combination of moderate- and vigorous-intensity activity) [8]. Therefore, increasing the number of children and adults that meet the recommended guidelines for physical activity has been a national target for public health efforts [9]. While estimates of physical activity among college-age students in Thailand are not currently known, it is estimated that only about 20% of Thai adolescents meet the recommended guidelines for physical activity [5]. It is also estimated that about 19.4% of the overall Thai population are physically inactive [10]. In response to low overall physical activity levels and high rates of sedentary behavior, the 1st National Conference on Physical Activity: “Active Living for All” was held in Bangkok in 2015, and a National Strategic Plan for Physical Activity was drafted the same year [11].

The use of electronic media (television, computers, cell phones and smartphones, etc.) or screen time has been cited as one of the main factors associated with sedentary lifestyles [12]. Despite the existence of a few studies investigating and tracking the physical activity of individuals from young adulthood to adulthood, college may be a time when students continue to cultivate physical activity and sedentary behaviors developed during childhood that they will carry throughout their lives [13]. The college-age years, which are often identified as “emerging adulthood,” are a time of transition from youth to the responsibilities of adulthood [14]. This is a period that is often overlooked by research despite the evidence that negative changes in physical activity and sedentary behaviors occur during this time [15]. Although college and university settings have been identified as targets for promoting these health-related behaviors, there is currently a lack of evidence to understand the relationship between physical activity and sedentary behaviors such as screen time [15].

Research has shown that college-age students in the US may spend an average of 8–10 h per day on a smartphone [16]. Similarly, there is evidence that Thailand has a higher rate of smartphone adoption, as well as the prevalence of internet and social media addiction (Facebook, Instagram, etc.), than most Asian countries [17]. Among Thai youth, more time is spent using social media applications on smartphones than is spent on productivity or work-related applications [17]. The association between screen time and physical activity is not well understood [18]. A commonly cited explanation for how screen time may impact physical activity is the displacement hypothesis, which postulates that the more time spent in sedentary behavior such as screen time, the less time that will be available for physical activity [19]. However, currently there is a lack of evidence to support the displacement hypothesis [18].

Although smartphone applications have been developed for tracking and monitoring health and physical activity, the evidence to support the use of smartphones and these applications to promote physical activity is conflicting [20,21]. There is also evidence to suggest that smartphone addiction exists in the college student population, and speculation that it can influence physical and mental health outcomes, such as obesity, poor physical ability, low self-esteem, increased sedentary behavior, and decreased social behavior [16,22,23]. Therefore, the college student population is an appropriate target for investigating determinants of physical activity participation, given the increased sedentary behaviors along with research indicating that 40–50% of college students are physically inactive and/or not meeting the recommended guidelines [24,25].

The purpose of the current study was to explore the amount of time spent using a smartphone and the physical activity levels of college students in health science-related majors from the US and Thailand, two countries with distinct cultural differences but have a similar fixation on using smartphones to inform intervention design within university settings. Despite distinct geographical, cultural, and economic differences, these two countries share similar public health issues related to low physical activity levels and high smartphone use. Therefore, the authors hypothesize that both US and Thai students that spend more time using a smartphone will have lower physical activity levels compared to students that spend less time using a smartphone.

## 2. Materials and Methods

This study was conducted with students enrolled at a university in Thailand from December 2015 to March 2016 and with students at a four-year university in the United States from August to November 2016. Each university’s institutional review board deemed the study met exemption status due to the involvement of minimal risk and the anonymous survey procedures.

### 2.1. Participants

Students were eligible to participate if they were 18 years of age or older. Using convenience sampling, students were recruited through an email sent to all students majoring in exercise science, nutrition, public health, and physical education based on the similarity of these majors at each institution, specifically the curriculum and requirements for graduation. The email invited students to complete an online survey about smartphone use and physical activity. Informed consent was obtained from participants on the first page of the online survey.

A total of 458 students completed the survey. Of the completed surveys, 15 were excluded due to missing data and 7 were excluded due to the student having an existing health condition that might impact their engaging in physical activity. This resulted in a final sample of 436 students included in the data analysis (*n*_US_ = 242; *n*_Thailand_ = 194).

### 2.2. Data Collection

The survey instrument was developed and conducted in Thailand and was then translated from Thai to English. Expert judges were consulted during survey development and survey items were inspected by a separate small group of college students to ensure item comprehension. The online surveys assessed participants’ self-reported age, race/ethnicity, height, weight, year in school, existing health conditions (congenital disease, chronic disease, injury, etc.), smartphone use, and physical activity levels. Students that reported any existing health conditions that might impact their ability to engage in physical activity were excluded from the analysis.

Current smartphone use was quantified through two questions that asked how many years participants have used a smartphone (never, less than 1 year, 1–3 years, 4–5 years, more than 5 years) and how much time per day they currently spend using their smartphone (never, less than 1 h per day, 1–3 h per day, 4–5 h per day, more than 5 h per day). To assess how participants use their smartphones, a series of questions asked if they currently use their smartphones for: communication such as “talking” and “messaging;” entertainment such as “internet,” “gaming,” “watching movies/TV,” “listening to music,” “taking and/or looking at photos,” “recording conversations/audio,” and “recording video;” social media such as “Facebook,” “Twitter,” and “Instagram;” and health/exercise such as “counting steps,” “jogging or running,” “counting calories consumed and expended,” “watching exercise videos,” “and “exercise planning.”

Physical activity levels of participants over the previous week were assessed through three questions. The first asked how many days per week on average participants engaged in physical activity (none, 1–2 days per week, 3–4 days per week, 5–6 days per week, 7 days per week). The second question asked the duration of physical activity during a typical session (10 min or less, 11–30 min, 31–45 min, 46–60 min, more than 60 min). The third question asked the intensity of physical activity during a typical session as either “light (minimal effort; usually you don’t sweat),” “moderate (not very tiring; could produce light sweat; can still talk to others),” or “vigorous (tiring; noticeably higher heartbeat; moderate to heavy sweating).”

### 2.3. Data Analyses

All statistical analyses were conducted using IBM SPSS Statistics for Mac, version 24 (IBM Corp., Armonk, NY, USA). Chi-square and *t*-tests were conducted to determine any significant differences in sex, age, and body mass index (BMI) between the countries. To determine if any differences existed in current use of smartphones, smartphone applications used, and physical activity between US and Thai students, Wilcoxon-Mann-Whitney and chi-square tests were conducted. Kruskal Wallis tests were conducted to determine students’ current use of smartphones by physical activity levels. The alpha level for these analyses was set at *p* < 0.05. Lastly, two-way ANOVAs with Bonferroni adjusted alpha levels of 0.025 per test (0.05/2) were conducted to examine the effects of country and categories of years using a smartphone on the number of days per week of physical activity and categories of time spent/day using a smartphone on the number of days per week of physical activity.

## 3. Results

About 78.0% of the participants were female and 22.0% were male. In terms of race/ethnicity, a majority of US students identified as white (97.1%) while students from Thailand all identified as Thai (100.0%). Most participants were 20–24 years of age (62.3%), followed by 15–19 years of age (26.1%) and 25–29 years of age (10.4%). The mean BMI for the total sample, the US, and Thailand were 23.3 kg/m^2^, 25.0 kg/m^2^, and 21.2 kg/m^2^, respectively. The demographic characteristics of the participants are provided in Table 1. The means and standard deviations of the variables related to smartphone use and physical activity are provided in Table 2.

Chi-square tests showed no significant difference in sex and age between the countries. The independent samples *t*-tests revealed that the two countries differed significantly on BMI with the US participants having a higher BMI than Thai participants (t(411)=8.82,p=0.000,d=0.87).

Wilcoxon-Mann-Whitney tests indicated no significant difference between the US and Thailand for the number of years using a smartphone (U=22,207.0,p=0.265); however, there was a significant difference in the amount of time using a smartphone per day with Thai participants reporting greater hours per day usage (*Mdn* = 4; 4–5 h/day) than US participants (*Mdn* = 3; 1–3 h/day), U=13,137.5,p=0.000,r=0.40. In terms of how participants used a smartphone, more US participants reported they used a smartphone for communication than did participants from Thailand, $X^{2}\left(1,N=439\right)=4.20,p=0.040,\varphi =0.10$, as well as for health and/or exercise, $X^{2}\left(1,N=439\right)=101.92,p<0.000,\varphi =0.48$. No difference was found between participants from the US and Thailand for using a smartphone for entertainment, $X^{2}\left(1,N=439\right)=0.48,p=0.488$, or social media, $X^{2}\left(1,N=439\right)=0.23,p=0.634$. The US participants reported more days per week of physical activity (*Mdn* = 3; 3–4 days/week) than participants from Thailand (*Mdn* = 2; 1–2 days/week), U=15,150.0,p=0.000,r=0.33, as well as greater duration of physical activity per session (*Mdn* = 4; 45–60 min) than participants from Thailand (*Mdn* = 3; 30–45 min), U=11,234.0,p=0.000,r=0.33. US participants also reported engaging in higher intensities of physical activity than participants from Thailand, $X^{2}\left(2,N=384\right)=84.22,p<0.000,\varphi =0.47$.

Among the US participants, Kruskal-Wallis tests showed that there was no significant difference in days per week of physical activity between the different categories of years of smartphone use, $X^{2}\left(2\right)=2.01,p=0.366$, and amount of time using a smartphone per day, $X^{2}\left(3\right)=2.39,p=0.496$. Among Thailand participants, there also was no significant difference in days per week of physical activity between the different categories of years of smartphone use, $X^{2}\left(3\right)=6.59,p=0.086$. However, a statistically significant difference was found among Thai participants in the days per week of physical activity between the different categories of time using a smartphone per day, $X^{2}\left(3\right)=10.55,p=0.014,\varepsilon^{2}=0.06$, with days per week of physical activity decreasing with increasing time using a smartphone per day.

A two-way ANOVA showed no significant interaction between the effects of country and categories of years using a smartphone on the number of days per week of physical activity, F(2,430)=1.73,p=0.178. There was also no significant interaction found between the effects of country and categories of time spent/day using a smartphone on the number of days per week of physical activity, F(3,429)=1.62,p=0.183.

## 4. Discussion

The study found that more US college students used smartphones for health and exercise as well as for communication than Thailand students. US college students were also more physically active than college students from Thailand in terms of number of days/week of physical activity, duration of time in each physical activity session, and the intensity of activity. College students from Thailand were found to use smartphones more per day than US students. No difference was found between US and Thailand college students in terms of the number of years using a smartphone. Greater smartphone use per day was found to be associated with less days per week of engaging in physical activity among the Thailand students, but not the US students.

Reasons for concern over the increased use of smartphones in college students relate to previous suggestions that sedentary behaviors in children and youth were associated with increased body fat percentage, decreased fitness and low self-esteem [22]. While our study did not find a relationship between PA and smartphone use among US students, there is still room for concern. Similar results regarding no relationship of smartphone use to physical activity was found in college students in a Midwestern US university with a variety of majors [23]. However, the authors were concerned with the fact that the time students spent on their smartphone was significantly related to increased sedentary behaviors. The authors also noted that when using a smartphone during physical activity the intensity of physical activity tended to be low, meaning that utilizing a smartphone while being active may coincide with reduced intensity of physical activity. Research supports the claim that use of a smartphone during physical activity may lead to reduced intensity of physical activity [26,27,28], however, evidence to support a causal relationship is lacking. Therefore, the concern then becomes if increased smartphone use leads to increased sedentary behavior, while simultaneously taking time away from physical activity and reducing the intensity of any physical activity. However, use of technology such as smartphones and apps during physical activity may have some benefits. Studies have shown that using smartphones may increase college students’ awareness of time spent in physical activity which may positively influence their overall physical activity levels [29]. Further research should investigate the potential relationships between smartphone use and time spent being sedentary, intensity of physical activity, and total daily physical activity.

The US students in the current study were more likely to use their smartphones for health and/or exercise, which may be a promising avenue for promoting physical activity through the development of smartphone apps specifically designed for higher education settings. The number of hours using smartphones per day among the Thai as well as US students also bodes well for this potential strategy. Indeed, research has shown that smartphone apps show promise for increasing physical activity among adults [29,30,31]. The design of these apps would be crucial, however, since as previously mentioned, the possible consequence of using a smartphone during physical activity may lower the intensity of the activity. The need for careful design of smartphone apps has been suggested due to the fact that most currently available apps for physical activity and exercise are lacking in the way they utilize evidence-based individual behavior change techniques [21]. While smartphone apps may provide some intrinsic motivation, research has suggested that incorporating technology into higher education settings should be done cautiously and deliberately by assessing the needs and resources of the institution as well as clearly defined objectives [32]. Given the potential for physical education and physical activity programs in colleges and universities to impact the student and faculty/staff population [33], it seems appropriate that smartphone apps be incorporated to enhance these programs and that they are designed at the institutional level to account for the college/university environments that impact individual behavior in order to improve sustainability [34]. It will be important for future smartphone app development to incorporate collaboration between app developers, college/university administrators and student health care providers, and local public health practitioners specializing in behavior change strategies in relation to physical activity.

College students were selected for this research as these are the years where lifetime physical activity habits can be established. In fact, research has found intention and habit to be significant predictors of physical activity in first generation college students [35]. While our sample included college students in health science-related majors, the emphasis on the importance of physical activity is noted; US students generally do participate in adequate physical activity while Thai students do not. This disparity may be due in part to differences in social/cultural and environmental factors among colleges/universities in the US and Thailand that influence physical activity.

On a policy level, the US has taken several steps to deal with the issue of low physical activity levels such as the establishment of the National Physical Activity Plan and the Physical Activity Guidelines for Americans [36,37]. However, despite the drafting of the National Strategic Plan for Physical Activity that lays out general recommendations, currently no comprehensive national strategic plan or established specific guidelines related to physical activity exist in Thailand [11,38]. Furthermore, the US National Physical Activity Plan specifically states that a strategy within the education sector is that “colleges and universities should provide students and employees with opportunities and incentives to adopt and maintain physically active lifestyles.” It will be important for Thailand to address specific strategies regarding universities within a comprehensive national strategic plan. These may include physical education opportunities for university students and environmental supports for active living. Specifically, in relation to the current study, the existence of these policies within the US may account for some of the difference seen in physical activity levels between US and Thai students.

The limitations of the study include recruitment from only health science-related disciplines and the use of self-reported measures of smartphone use and physical activity. Students in health science-related disciplines most likely have a greater understanding of physical activity and sedentary behavior as well as the recommended guidelines for physical activity. Indeed, students in these majors at both of the study universities are required to learn about physical activity and sedentary behaviors. This may lead them to be more physically active than non-health science-related majors. But, in addition, recent research has found that adults tend to overreport their physical activity [39] and underreport their smartphone use [40]. Therefore, these students may be more likely to overreport their physical activity levels and underreport their use of smartphones as a form of social desirability. A further limitation is that the study sample was predominantly female and while there is conflicting evidence on gender differences in sedentary behavior and screen time, research consistently indicates that female college students tend to be less physically active than their male counterparts [41,42]. Lastly, this study was conducted at only one university in each country and therefore generalizability of the results is limited. However, the authors are not aware of any research examining distinctly different countries that share similar issues around excessive smartphone use and physical activity levels and therefore the current study is an initial step in examining a potential global problem.

## 5. Conclusions

The higher smartphone use among college students, especially as indicated in Thailand, may be a barrier as well as a strategy for physical activity. Carefully designed smartphone applications (fitness apps, running apps, etc.) specifically targeting students in settings of higher education have the potential to promote physical activity, however, current research is conflicting. As smartphone use is likely to only increase in the future, research should examine the best techniques for smartphone application development in colleges and universities to promote physical activity among students.

## Figures and Tables

**Table 1:** Demographic characteristics of US and Thailand participants *.

Variable	US	Thailand
Total	242	194
Sex [*n* (%)]		
Female	194 (80.2)	146 (75.3)
Male	48 (19.8)	48 (24.7)
Race/ethnicity [(*n* (%)]		
White	235 (97.1)	0 (0.0)
Black/African American	7 (2.9)	0 (0.0)
Thai	0 (0.0)	194 (100.0)
Age group [*n* (%)]		
15–19 years	70 (28.6)	40 (20.6)
20–24 years	134 (54.7)	129 (66.5)
25–29 years	19 (7.8)	25 (12.9)
30 years or older	5 (2.0)	0 (0.0)
Body mass index [*M* (*SD*)]	25.0 (4.80)	21.2 (4.24)
Female	24.3 (4.53)	20.9 (4.62)
Male	27.6 (5.04)	22.0 (2.67)

* Some variables do not sum to 100 due to missing data.

**Table 2:** Means, standard deviations, and percentages for smartphone use and physical activity.

Variable	US	Thailand
Number of years using a smartphone * [*M* (*SD*)]	3.25 (0.74)	3.12 (0.89)
Average time using a smartphone per day ** [*M* (*SD*)]	2.75 (0.74)	3.40 (0.78)
Smartphone applications used *** [*n* (%)]		
Communication	243 (99.2)	187 (96.4)
Entertainment	241 (98.4)	189 (97.4)
Social media	237 (96.7)	186 (95.9)
Health/exercise	183 (74.7)	51 (26.3)
Exercised in past month *** [*n* (%)]	223 (91.0)	161 (83.0)
Days per week of physical activity ^#^ [*M* (*SD*)]	2.92 (0.97)	2.31 (0.99)
Duration of physical activity per session ^##^ [*M* (*SD*)]	3.71 (0.95)	2.93 (1.21)
Intensity of physical activity per session ^###^ [*M* (*SD*)]	2.52 (0.54)	1.98 (0.47)

Note: * 5-point scale from 1 (never) to 5 (more than 5 years); ** 5-point scale from 1 (never) to 5 (more than 5 h); *** Dichotomous variable: 1 (no), 2 (yes); ^#^ 5-point scale from 1 (none) to 5 (7 days); ^##^ 5-point scale from 1 (less than 10 min) to 5 (more than 60 min); ^###^ 3-point scale from 1 (light) to 3 (vigorous).
